# Characterization of the role of sodium nitroprusside (SNP) involved in long vase life of different carnation cultivars

**DOI:** 10.1186/s12870-017-1097-0

**Published:** 2017-09-06

**Authors:** Aung Htay Naing, Kyoungsun Lee, Muthukrishnan Arun, Ki Byung Lim, Chang Kil Kim

**Affiliations:** 1 Department of Horticultural Science, Kyungpook National University, Daegu, 4165122 South Korea; 2 Department of Biotechnology and Genetic Engineering, School of Biotechnology, Bharathidasan University, Tiruchirappalli, Tamil Nadu 620024 India

**Keywords:** ACC, Antioxidant activity, Ethylene production, Relative fresh weight, Relative gene expression

## Abstract

**Background:**

Sodium nitroprusside (SNP) has been previously shown to extend the vase life of various cut flowers; however, its positive effect on extending vase life of carnations has not been well documented. Moreover, the role of SNP in the mechanisms underlying determination of vase life of cut carnations has also not been well addressed.

**Results:**

SNP increased vase life of Tico Viola carnations along with their relative fresh weight (RFW). Among the treatments, the flowers treated with 10 mg L^−1^ SNP had the longest vase life and maximum relative fresh weight (RFW). This was achieved through significant suppression of ethylene production via downregulation of ethylene biosynthesis and petal senescence-related genes, and through an increase in the scavenging mechanism of reactive oxygen species (ROS) by antioxidant activity during flower vase life. In addition, the positive efficacy of SNP could also be confirmed using 1-aminocyclopropane-1-carboxylic acid (ACC) and different cultivars, resulting in similar trends for both experiments.

**Conclusion:**

Taken together, these results suggest that SNP plays a crucial role in multiple modes of action that are associated with the longevity of cut carnation flowers.

**Electronic supplementary material:**

The online version of this article (10.1186/s12870-017-1097-0) contains supplementary material, which is available to authorized users.

## Background

Carnations (*Dianthus caryophyllus* L.) are one of the most important ornamental plants, and are widely used as a cut flower and a bedding plant in gardens [1]. However, when used as a cut flower, carnation postharvest senescence occurs within a week for most cultivars, which is a major challenge in marketing of the cut flowers. As carnation flowers are highly sensitive to ethylene [2], their postharvest life likely depends on their endogenous ethylene production. To extend the postharvest life of cut flowers, researchers have made considerable efforts using various chemicals [3, 4]. Generally, silver thiosulfate (STS) is widely used to delay postharvest senescence because it can serve as an inhibitor of ethylene action [3, 5, 6]. However, recently, there have been major concerns about potential contamination of the environment due to waste STS solutions [7]. 1-methylcyclopropene (1-MCP), an environmentally-friendly ethylene inhibitor, has been found to extend the postharvest life of carnations by inhibiting ethylene production [3, 4, 8–10]. Moreover, nitric oxide (NO), which is also an environmentally acceptable compound, has been shown to be effective in extending the postharvest life of various cut flowers including carnations [3, 11]. However, the gaseous nature of both compounds is a hurdle to their commercial usage [3].

Recently, there has been great interest in the application of sodium nitroprusside (SNP), an NO donor, to extend the vase life of cut flowers; its positive effects for elongating the postharvest life have been demonstrated in various cut flowers such as gladiolus and rose [12–14]. However, there has been only one report describing the role of SNP in the postharvest life of carnations [15]. Dwivedi et al. [14] recently claimed that SNP can increase the postharvest life of gladiolus flowers by downregulating senescence-associated genes as well as by enhancing antioxidant activity. Liao et al. [13] observed that SNP enhanced the postharvest life of cut roses by inhibiting 1-aminocyclopropane-1-carboxylate oxidase (ACO) activity involved in ethylene production. Tanase et al. [16] reported that long postharvest life of cut carnation flowers is associated with low expression of ACC synthase and ACC oxidase genes (*ACS1, ACS2,* and *ACO1*) in the gynoecia and petals.

However, in carnations treated with SNP, Zeng et al. [15] reported the role of SNP in prolonging the postharvest life of cut flowers by showing an enhancement in antioxidant activity only. To date, there is little data on how SNP extends the vase life of cut flowers, especially in carnations. In addition, there have been no investigative experiments on ethylene production, variation in ethylene production-related genes, and reactive oxygen species (ROS)-scavenging activity in response to SNP.

Hence, it is interesting to study how SNP relates to the above-mentioned parameters that are involved in the process of flower senescence. Therefore, we tried to investigate the role of SNP in the process of cut flower senescence by determining the flower senescence-associated parameters such as ethylene production, expression of ethylene production-related genes, and variation in antioxidant activity during flower vase life.

## Methods

### Plant material

Cut flowers of carnations (Tico Viola), graded for marketable quality, were obtained from a local flower production farm located 30 km away from the laboratory. Upon arrival in the laboratory, the cut flower stems were re-cut to a length of approximately 40 cm, in accordance with commercial practices. In addition, the leaves on the stems that would be submerged in vase water were carefully removed by hand.

### Treatment with SNP

Glass bottles (500 mL volume) were used as vases for this experiment; they were filled with 250 mL distilled water containing different concentrations of sodium nitroprusside (SNP) (Enzo Life Sciences) [0 (control), 1, 5, 10, 15, or 20 mg L^−1^]. SNP stock solution was prepared following the manufacturer’s instructions. Five cut stems, which had approximately the same fresh weight, were placed into the vases filled with different concentrations of SNP for 24 h. To avoid photodegradation of SNP (releasing nitrosyl ligand and cyanide ion), the vases were wrapped with aluminum foil during the SNP treatment. Next, the treated stems were thoroughly washed under tap water and replaced into vases containing 250 mL distilled water. The vases were then maintained in a growth chamber at a light intensity of 20 μmol^−2^ s^−1^ for 12 h, at 23 °C and 60–70% relative humidity. There were three vases (15 flowers) per treatment and the experiment was conducted three times.

The initial fresh weight of all flowers in each vase was recorded, after which five flowers were selected to evaluate the relative fresh weight and vase life throughout the experiment. The remaining 10 flowers were used for the estimation of ethylene production, transcriptional analysis of ethylene biosynthesis genes (*DcACO1* and *DcACS1*) and petal senescence-related gene (Cysteine Proteinase Inhibitor; *CPI* gene), and antioxidant activity measurement using traits such as 1, 1-diphenyl-2-picrylhydrazyl (DPPH), 2, 2′-azino-bis-3-ethylbenzthiazoline-6-sulphonic acid (ABTS), total polyphenol, and total flavonoid content.

### Treatment with ACC and SNP

In the above experiment, 10 mg L^−1^ was found to be the optimal concentration for ethylene inhibition and long vase life of the flowers. To confirm the role of the SNP concentration, 5 cut stems with approximately the same fresh weight, as used in the above experiment, were placed into the same vase containing 250 mL distilled water (control) with ethylene precursor ACC 1 mg L^−1^ (Sigma Aldrich), either alone or in combination with 10 mg L^−1^ SNP. The vases were then maintained in the same growth chamber that was used in the above experiment, and the physiological and molecular analyses from the above experiment were performed again for this experiment. There were three vases (15 flowers) per treatment and the experiment was conducted three times.

### Effect of SNP on postharvest life of different genotypes

SNP at 10 mg L^−1^ concentration was found to improve the postharvest life of the carnation Tico Viola; however, we were interested in determining whether SNP would have the same effects on other cultivars, such as Venus, Tico Tico, and Shino Lily. Hence, these other cultivars were obtained from the same flower production farm where Tico Viola was grown. Their samples were prepared in the same manner as done for Tico Viola, and 5 cut stems were placed into the same vase containing 250 mL distilled water (control) with 10 mg L^−1^ SNP. The vases were then maintained in the same growth chambers that were used in the above experiment. In addition, the same physiological and molecular analyses from the above experiments were performed in this experiment. There were three vases (15 flowers) per treatment for each cultivar and the experiment was conducted three times.

### Postharvest life and relative fresh weight (RFW)

Postharvest life of each flower was determined when more than one-third of its petals showed in-rolling, browning, or loss of ornamental value. The fresh weight of each cut stem was measured daily, and the relative fresh weight (RFW) was calculated using the formula: RFW (%) = (FWt/FW0) × 100; where FWt is the fresh weight of the stem (g) at day (3, 6, or 9) and FW0 is the initial fresh weight of stem (g) at day 1 [17]. Five flowers per treatment were used with three replications.

### Ethylene measurements

For ethylene measurements, petals (5 g) from each treatment were weighed and sampled after different periods (days 3, 6, and 9). They were placed in a 50-mL glass tube and enclosed with a rubber septum for 16 h at 20 °C. An aliquot of the accumulated gas (1 mL) was withdrawn using a 1-mL syringe through the septum and analyzed for ethylene using a gas chromatograph (GC-2010, Shimadzu). Three syringes (three replicates) were used for each treatment.

### RNA extraction and quantitative real time PCR (qRT-PCR) analysis

Total RNA was extracted from 100 mg of petals using the RNeasy Plant Mini Kit (Qiagen, Hilden, Germany). The cDNA was synthesized from 1 μg of the total RNA with an oligo dT_20_ primer using a reverse transcription kit (ReverTra Ace-á, Toyobo, Japan). Transcript levels of ethylene production-related genes (*DcACO1* and *DcACS1*), and petal senescence-related gene (cysteine proteinase inhibitor gene; *DcCPI*) were measured using a StepOnePlus Real-Time PCR system (Thermo Fisher Scientific, Waltham, USA) [18]. To confirm the amount of template RNA, a fragment of carnation actin (*DcACT*) was used as the internal control. The primers and PCR conditions for the detected genes are listed in (Additional file 1: Table S1). Three samples per treatment were used, and the analysis was repeated three times.

### Determination of antioxidant activity

Petals were collected from the flowers on day 9 after treatment, when most of the control flowers showed a loss in their ornamental value, and were frozen for analysis of antioxidant activity.

For DPPH and ABTS activity, 5 g of frozen petals were used and the analyses were performed following the methods of Kim et al. [19]. For the total polyphenol and total flavonoid content, we followed the methods of Dewanto et al. [20]. There were three samples per treatment, and the analysis was repeated three times.

### Statistical analysis

Data were analyzed in SPSS version 11.09 (IBM Corporation, Armonk, USA) and are presented as means of three replicates. The significance differences among the means were analyzed at *P* < 0.05 or 0.01.

## Results

### Vase life, ethylene production, and relative fresh weight (RFW)

**Fig. 1:**
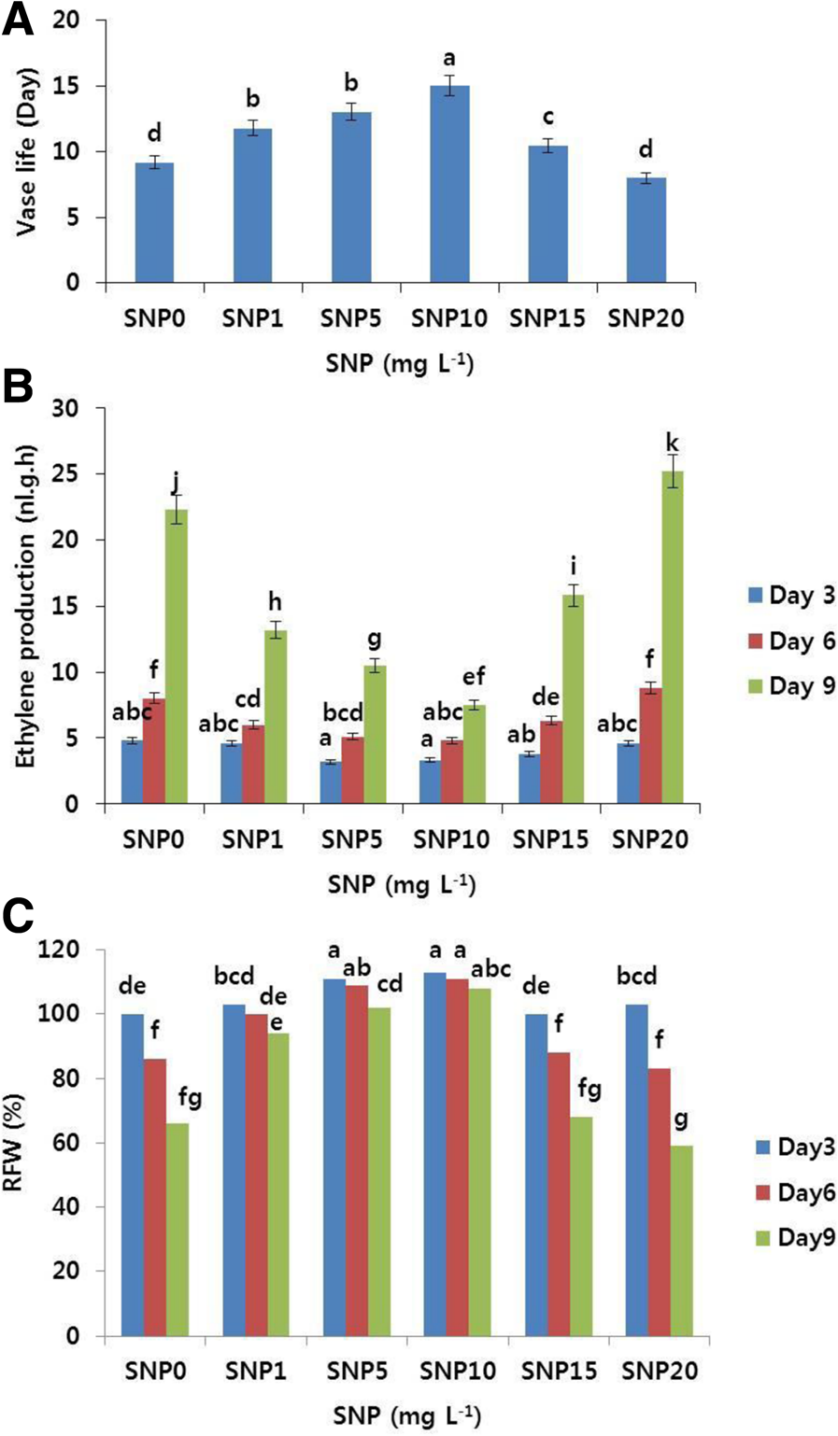
Effects of different concentrations of sodium nitroprusside (SNP) on the vase life (**a**), ethylene production (**b**), and relative fresh weight (RFW) (**c**) of cut carnation flowers (‘Tico Viola’) during the vase life period. Data represent mean of three replicates, while bar indicates standard deviation. Means with different letters are significantly different (Tukey’s HSD test, *p* < 0.05)

The vase life of cut carnation flowers (Tico Viola) responded differently to SNP, and the effects were dose-dependent (Additional file 1: Figure S1). Based on the results shown in Fig. 1a, most of the treatment concentrations extended the vase life compared with the control, whereas the vase life of flowers treated with 20 mg L^−1^ SNP declined. Specifically, at 10 mg L^−1^, SNP significantly extended the vase life by approximately 6 days, and there was an increase of 2.6, 3.8, and 1.6 days at SNP concentrations of 1, 5, and 15 mg L^−1^, respectively, compared with the control. Thus, 10 mg L^−1^ was considered the optimal SNP concentration and used for further experiments.

Generally, the vase life of cut flowers was associated with their ethylene production during the vase life period. For the control and SNP-treated (1, 15, 20 mg L^−1^) cut flowers, the ethylene production rate was relatively low at day 3; however, it began to periodically increase and tended to reach a peak by day 9 (Fig. 1b). In fact, the ethylene production rate of the flowers at day 9 after the treatment was observed to be the highest in control and 20 mg L^−1^ SNP treatments, showing petal in-rolling (sign of senescence) on days 9 and 8 after the treatment, followed by ethylene production in SNP treatments at 15 and 1 mg L^−1^, respectively. In case of other SNP treatments (5 and 10 mg L^−1^), the ethylene production rate increased markedly on day 9 and further increments were noticed until day 12 (data not shown); however, the ethylene production rate in 10 mg L^−1^ SNP treatment seemed to increase until day 15, when the flowers finally showed petal in-rolling. Overall, the ethylene production rate was likely to be associated with the symptoms of flower senescence such as petal in-rolling and wilting.

Normally, RFW is also strongly associated with the vase life of cut flowers. In this study, the changes in RFW of cut carnation flowers exhibited similar trends in both control and SNP treatments. RFWs were the highest on day 3 for the control and some of the SNP treatments (1, 15, and 20 mg L^−1^), and decreased thereafter (Fig. 1c). Similarly, 5 and 10 mg L^−1^ SNP treatments also gave the highest RFWs on day 3; whereas the RFWs of 10 mg L^−1^ SNP were not significantly different between days 3 and 6, but they declined thereafter, in fact, the RFW of 10 mg L^−1^ SNP treatment on day 9 was still higher than that of 5 mg L^−1^ SNP treatment (Fig. 1c). Throughout the vase life period, the RFWs of 10 mg L^−1^ SNP treatment were significantly higher than that of the other treatments.

### Quantification of the genes related to ethylene production and flower senescence

**Fig. 2:**
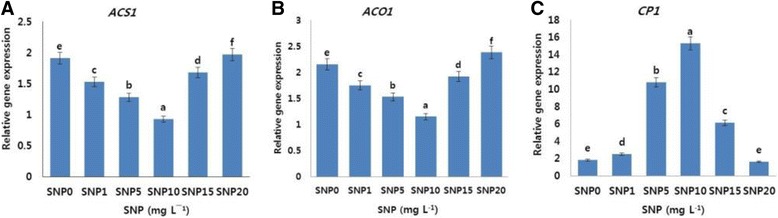
Effects of different concentrations of sodium nitroprusside (SNP) on the transcript levels of ethylene biosynthesis genes (*DcACS1* (**a**), *DcACO1*) (**b**), and petal senescence gene (*DcCPI*) (**c**) in the petals of ‘Tico Viola’, which were collected on day 9 after the treatment. Data represent mean of three replicates, while bar indicates standard deviation. Means with different letters are significantly different (Tukey’s HSD test, *p* < 0.05)

To understand the relationship between ethylene production and the expression level of ethylene biosynthesis or receptor genes, the transcript levels of the ethylene biosynthesis (*DcACS1* and *DcACO1*) genes were determined on day 9. The expression profiles of the ethylene biosynthesis genes are presented in Fig. 2a and b. As expected, the transcript levels of the detected genes expressed in the flowers on day 9 were the highest in the control and 20 mg L^−1^ SNP treatment, followed by other SNP treatments (15, 1 and 5 mg L^−1^), whereas the lowest expression levels were noted in the 10 mg L^−1^ SNP treatment. These findings support the conclusion that ethylene production was strongly associated with ethylene-related gene expression.

Unlike the ethylene production-related genes, the expression and transcript levels of *DcCPI* were observed to be the highest in the 10 mg L^−1^ SNP treatment, followed by the 5, 15, and 1 mg L^−1^ treatments, whereas the lowest levels were observed in the control and 20 mg L^−1^ SNP treatments (Fig. 2c). These findings indicated that *DcCPI* plays important role in the petal senescence of cut carnation flowers.

### Antioxidant activity

**Fig. 3:**
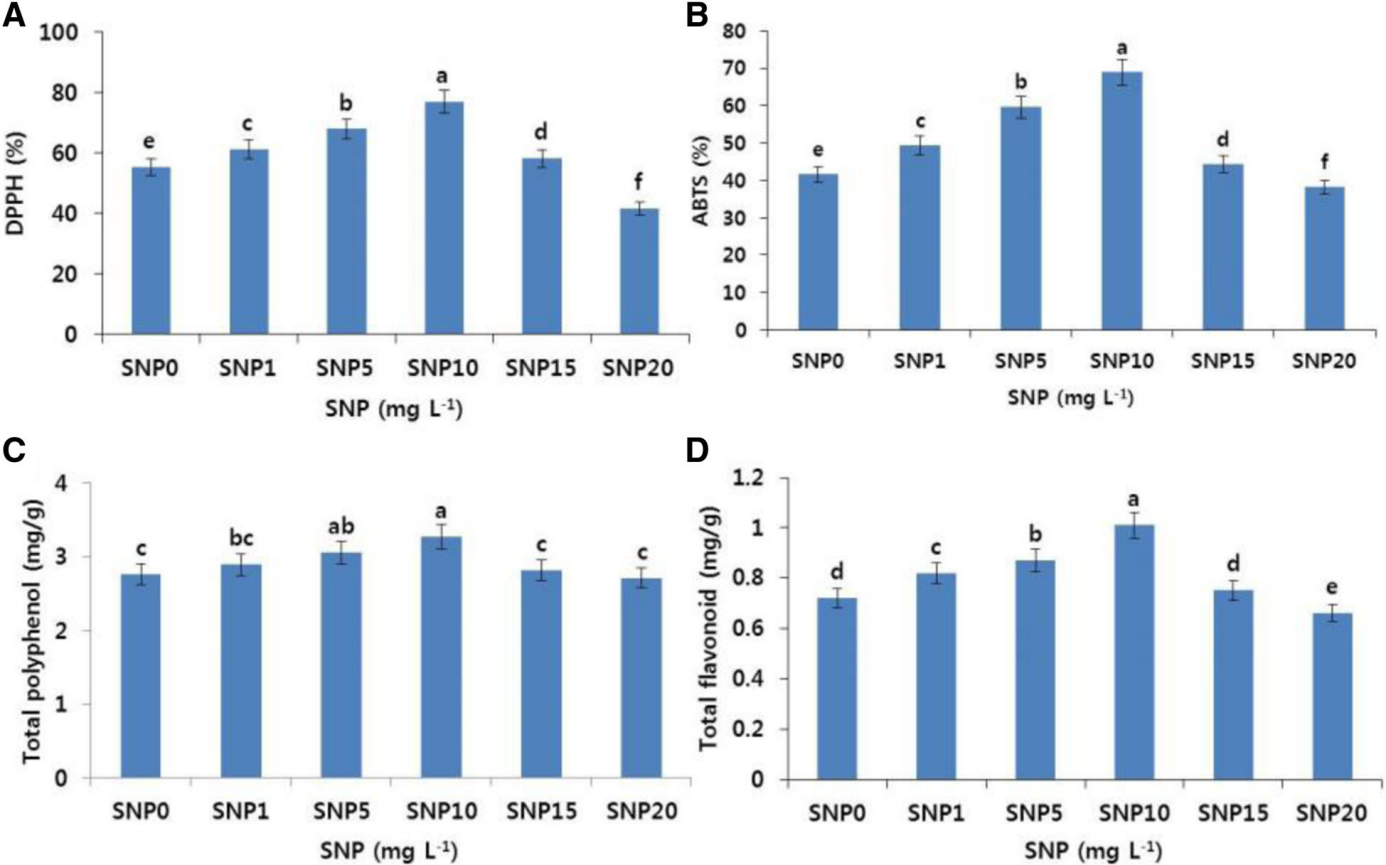
Effects of different concentrations of sodium nitroprusside (SNP) on DPPH activity (**a**), ABTS activity (**b**), total polyphenol content (**c**), and total flavonoid content (**d**) in the petals of ‘Tico Viola’, which were collected on day 9 after treatment. Data represent mean of three replicates, while bar indicates standard deviation. Means with different letters are significantly different (Tukey’s HSD test, p < 0.05)

In the Tico Viola carnations, petal senescence in the control flowers was observed on day 9, whereas SNP treatments extended the vase life of the flowers. Thus, on day 9, we determined the ROS-scavenging activity using DPPH and ABTS assays, and the total polyphenol and total flavonoid content of the flowers. The ROS-scavenging activity in all SNP treatments except 20 mg L^−1^ was significantly higher than in the control (Fig. 3a and b). Specifically, the activity was the highest in 10 mg L^−1^ SNP treatment followed by the other SNP treatments (5 > 1 > 15 mg L^−1^). Additionally, the antioxidant activity profiles (for total polyphenol and flavonoid) for both the control and the SNP treatments were also similar to those of ROS-scavenging activity (Fig. 3c and d). From the findings, it is obvious that SNP extends vase life of carnations by enhancing the antioxidant activity and reducing the transcript levels of the genes involved in ethylene production and petal-senescence.

### Treatment with ACC

**Fig. 4:**
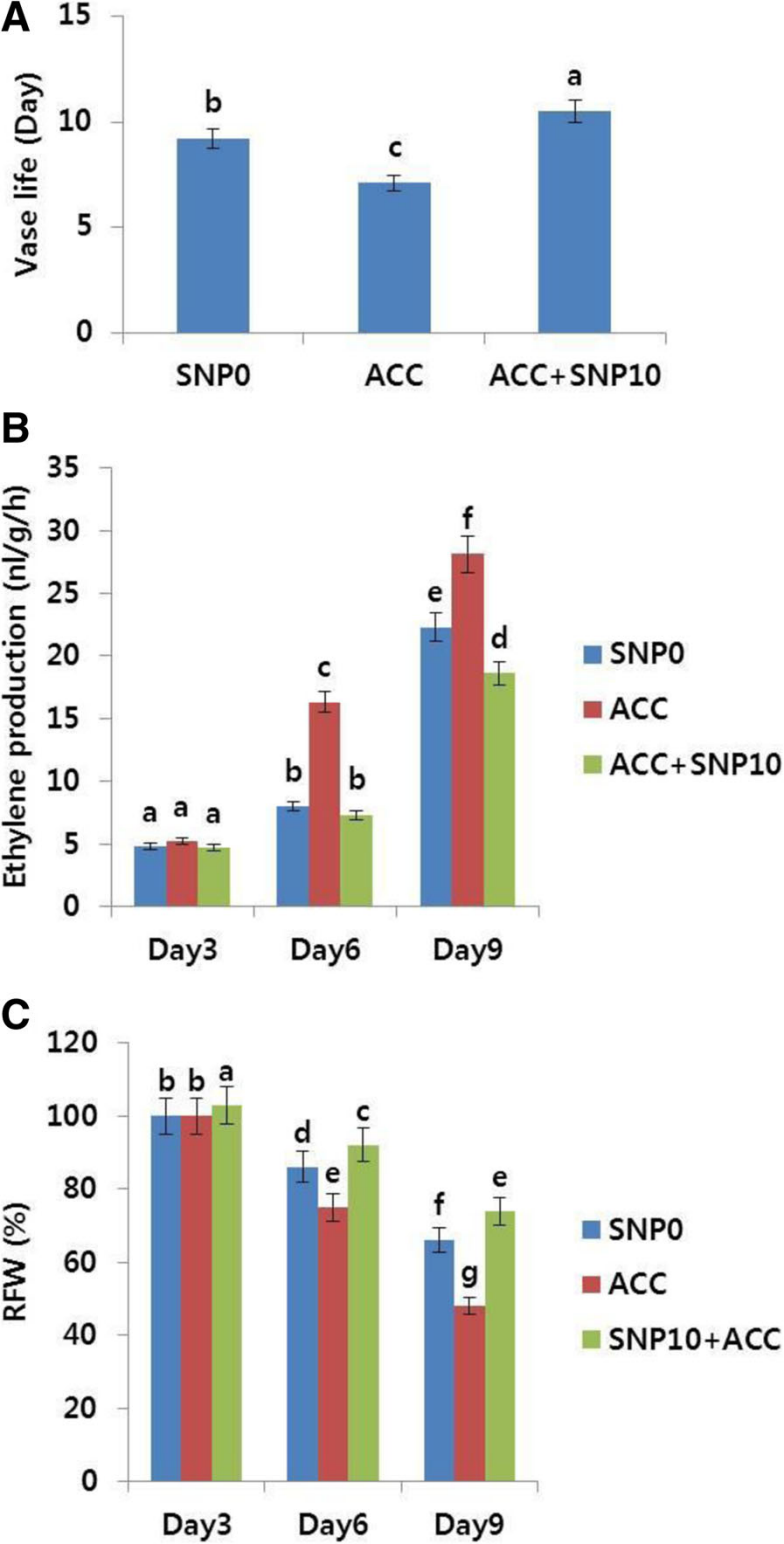
Effects of 1-aminocyclopropane-1-carboxylic acid (ACC; 1 mg L^−1^) alone and in combination with sodium nitroprusside (ACC; 1 mg L^−1^ + SNP; 10 mg L^−1^) on the vase life (**a**), ethylene production (**b**), and relative fresh weight (RFW) (**c**) of cut carnation flowers (‘Tico Viola’) during the vase life period. Data represent mean of three replicates, while bar indicates standard deviation. Means with different letters are significantly different (LSD test, p < 0.05)

It was of interest to confirm the effect of SNP treatment on the vase life of cut flowers in combination with an ethylene precursor (ACC). When ACC was added to the vase solution (distilled water), flowers showed obvious signs of senescence (petal in-rolling) by day 7 (Additional file 1: Figure S2) and the vase life was found to be 2 days shorter than that of the controls; however, addition of ACC to the vase solution containing 10 mg L^−1^ SNP extended the vase life by 1.3 days over the controls (Fig. 4a). Although ACC inhibited the vase life of the flowers, when it was combined with 10 mg L^−1^ SNP, the vase life of the flowers was still longer than that in the control. Thus, this result supports a positive effect of SNP on the vase life of cut carnation flowers.

Moreover, the ethylene production rate in the flowers treated with ACC alone rapidly increased and was higher than that in control, particularly on days 6 and 9, whereas the production on day 6 between control and the combination (ACC and SNP) was not significantly different (Fig. 4b). However, the ethylene production rapidly increased on day 9 and vase life of the flowers also ended after day 10. In addition, the RFW obtained for ACC was also lower than control on days 6 and 9 (Fig. 4c). When ACC was added to the SNP-containing solution, a distinct increase in RFW was noted.

**Fig. 5:**
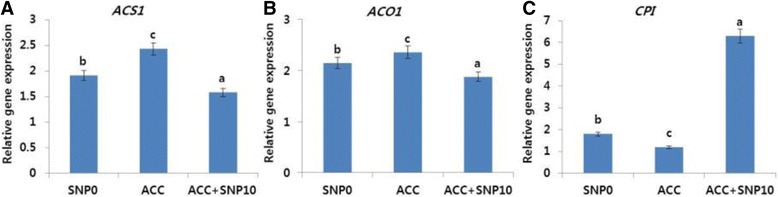
Effects of 1-aminocyclopropane-1-carboxylic acid (ACC; 1 mg L^−1^) alone and in combination with sodium nitroprusside (ACC; 1 mg L^−1^ + SNP; 10 mg L^−1^) on the transcript levels of ethylene biosynthesis genes (*DcACS1* (**a**), *DcACO1*) (**b**), and petal senescence gene (*DcCPI*) (**c**) in the petals of ‘Tico Viola’, which were collected on day 9 after the treatment. Data represent mean of three replicates, while bar indicates standard deviation. Means with different letters are significantly different (LSD test, p < 0.05)

In response to ACC addition, the vase life of the cut flowers was shortened by increased ethylene production. Thus, the transcript levels of the ethylene production-related genes and the petal senescence-related genes were determined in the ACC-treated flowers along with controls. As expected, the transcript levels of *DcACO1* and *DcACS1* genes were significantly higher in ACC treatment than in controls, whose transcript levels were, in turn, higher than those in the ACC + SNP treatment (Fig. 5a and b). In addition, the transcript level of the petal senescence-related gene (*DcCPI*) expressed in flowers treated with ACC + SNP was also higher than in both controls and flowers treated with ACC, whereas the transcript levels were higher in controls than in ACC treatment (Fig. 5c).

**Fig. 6:**
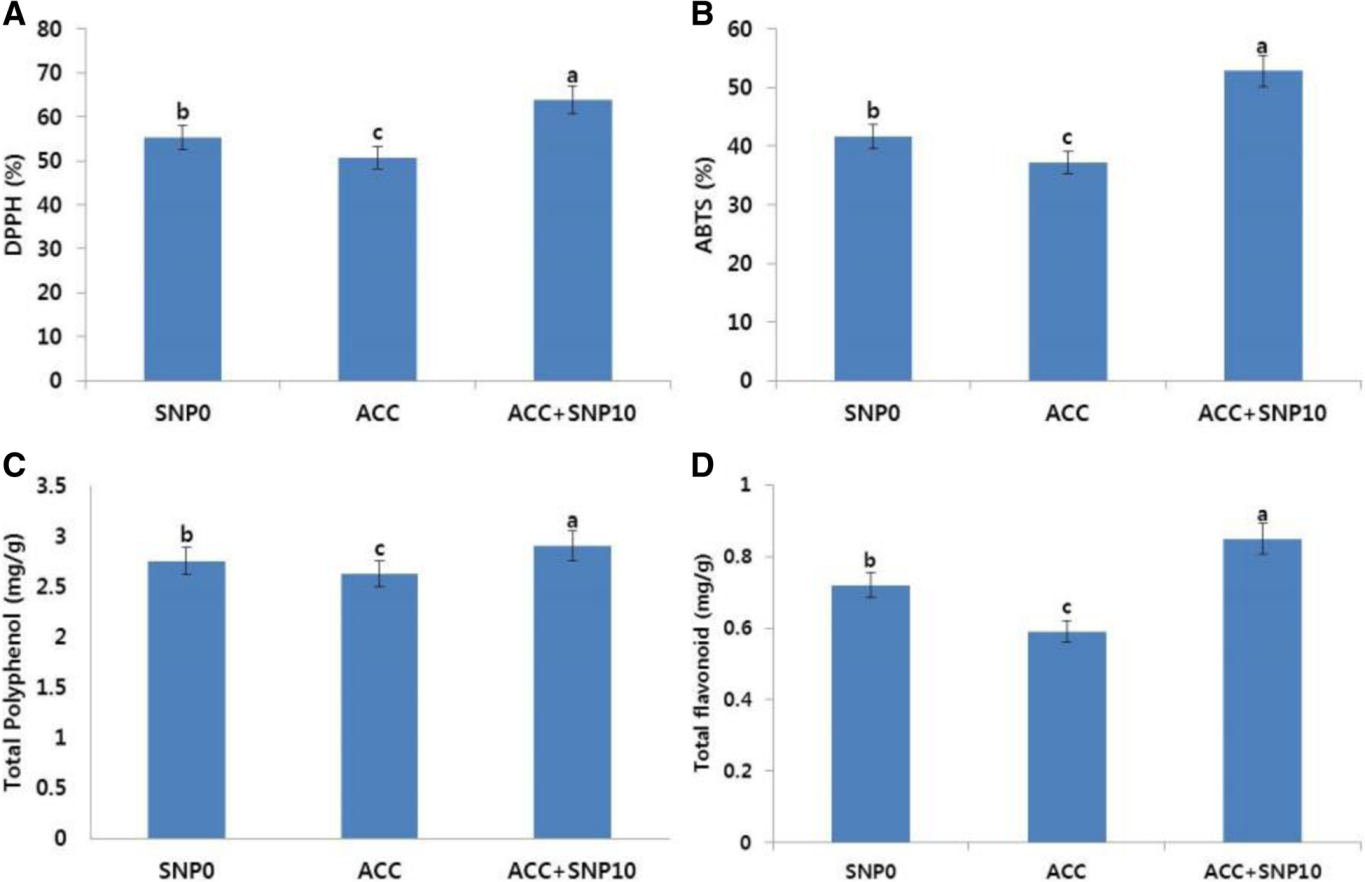
Effects of 1-aminocyclopropane-1-carboxylic acid (ACC; 1 mg L^−1^) alone and in combination with sodium nitroprusside (ACC; 1 mg L^−1^ + SNP; 10 mg L^−1^) on DPPH activity (**a**), ABTS activity (**b**), total polyphenol content (**c**), and total flavonoid content (**d**) in petals of ‘Tico Viola’, which were collected at day 9 after treatment. Data represent mean of three replicates, while bar indicates standard deviation. Means with different letters are significantly different (LSD test, p < 0.05)

Moreover, we also determined the ROS-scavenging activity (DPPH and ABTS activity) and antioxidant activity (total polyphenol and flavonoid content) in the flowers treated with ACC, to examine whether the activity was reduced earlier than in the controls. Results shown in Fig. 6 indicate that the ROS-scavenging activity in the ACC treatment was lower than that in controls; however, the activity increased when SNP was combined with ACC. Similarly, the total polyphenol and flavonoid content detected in ACC was also lower than that in controls, but SNP addition enhanced this activity. These findings suggest that SNP has the ability to extend the vase life of flowers even when present in combination with ACC, by increasing the antioxidant activity, and by reducing the transcript levels of ethylene production-related and petal senescence-related genes.

### Effect of SNP on vase life of different genotypes

**Fig. 7:**
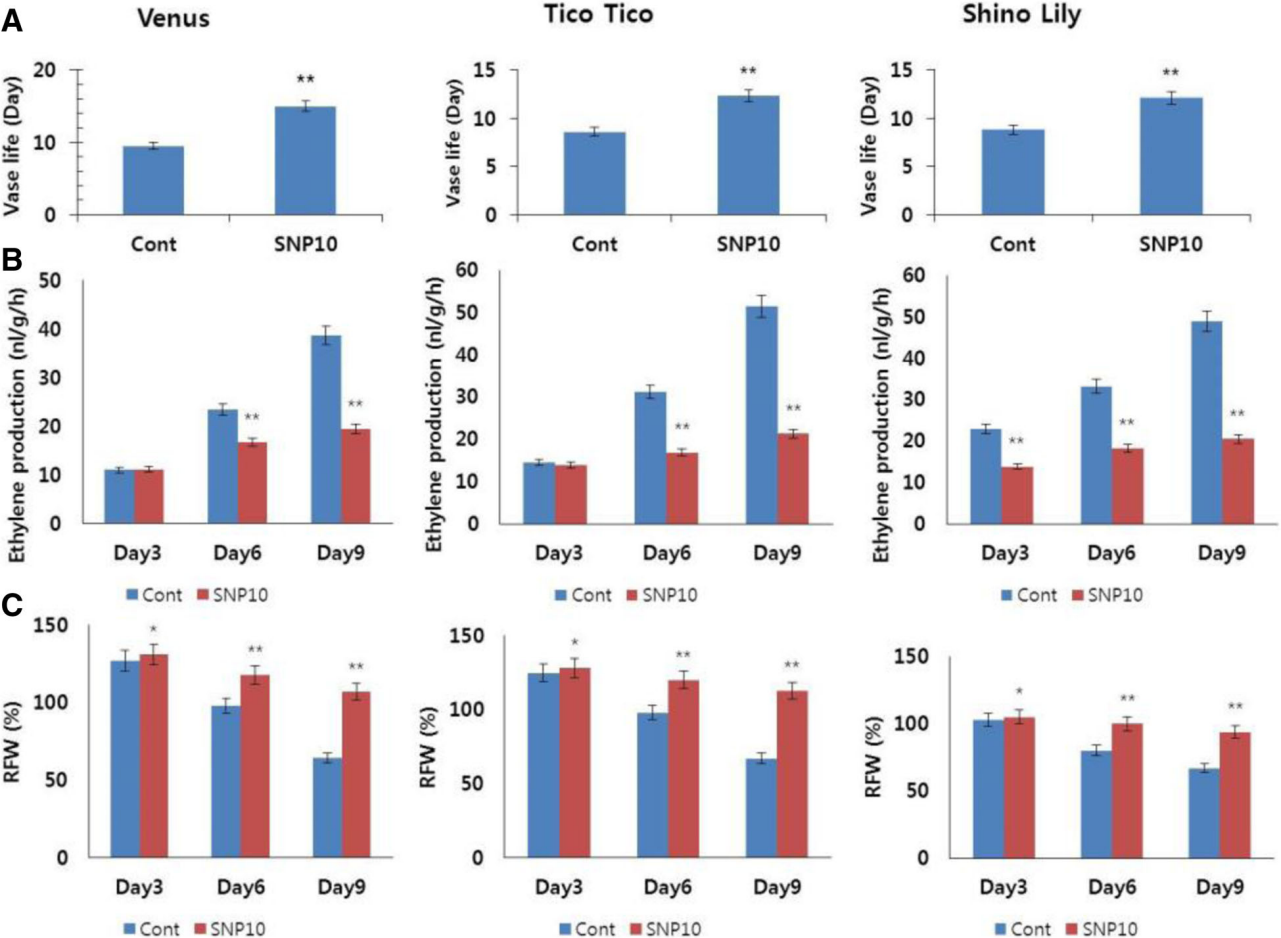
Effects of sodium nitroprusside (SNP; 10 mg L^−1^) on the vase life (**a**), ethylene production (**b**), and relative fresh weight (RFW) (**c**) of different cut carnation flowers (‘Venus’, ‘Tico Tico’, ‘Shino Lily’) during the vase life period. Data represent mean of three replicates, while bar indicates standard deviation. Means with asterisk(s) are statistically significant (T-test, **p < 0.01, *p < 0.05)

We designed experiments to confirm the role of this SNP concentration (10 mg L^−1^) in different carnation cultivars, i.e., Venus, Tico Tico, and Shino Lily. As shown in Fig. 7a, the length of the flower vase life did not differ significantly among the tested cultivars; however, the vase life of Venus was 9 days, one day longer than that of the other two cultivars. When 10 mg L^−1^ SNP was used, the vase life of all cultivars improved. The vase life was 15 days for Venus and 12 days for Tico Tico and Shino Lily, resulting in an improvement of 4 days for the cultivars Tico Tico and Shino Lily, and 6 days for Venus, compared with the respective controls (Fig. 7a and Additional file 1: Figure S3). In addition, ethylene production by all cultivars was significantly higher in the controls than in SNP treatments (Fig. 7b); ethylene production for controls started increasing markedly on day 6 and reached a peak by day 9, when most of the vase lives ended. Thus, we predicted that the peak of ethylene production rate for treatments would be within days 12–15 because the cultivars’ vase lives ended around this period. RFWs for all the cultivars reached the highest values during the first 3 days after treatment, for both controls and treatments, and it declined thereafter; a quick decrease was observed in the controls, whereas a slow decrease was noted in the treatments (Fig. 7c).

**Fig. 8:**
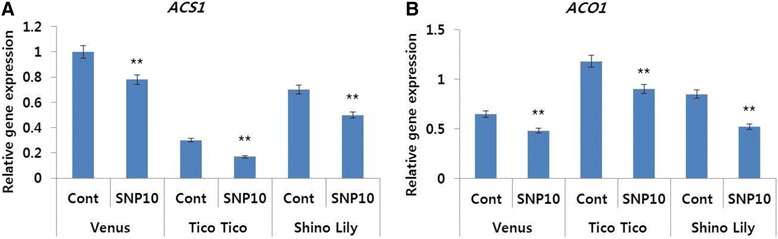
Effect of sodium nitroprusside (SNP; 10 mg L^−1^) on the transcript levels of ethylene biosynthesis genes (*DcACS1* (**a**) and *DcACO1)* (**b**), in the petals of different carnation ‘Venus’, ‘Tico Tico’, and ‘Shino Lily’, which were collected on day 9 after the treatment. Data represent mean of three replicates, while bar indicates standard deviation. Means with two asterisks (**) are highly significant (T-test, p < 0.01)

**Fig. 9:**
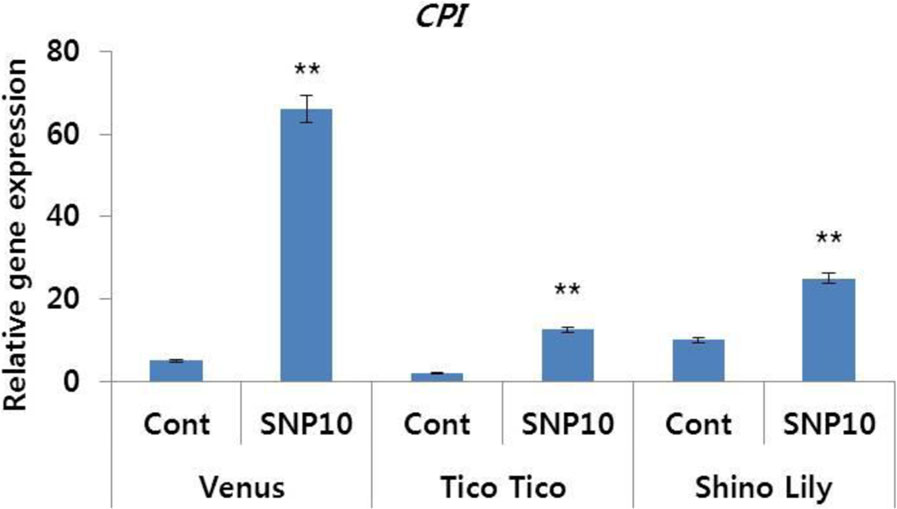
Effects of sodium nitroprusside (SNP; 10 mg L^−1^) on the transcript level of petal senescence gene (*DcCPI*) in the petals of different carnation ‘Venus’, ‘Tico Tico’, and ‘Shino Lily’, which were collected on day 9 after treatment. Data represent mean of three replicates, while bar indicates standard deviation. Means with two asterisks (**) are highly significant (T-test, p < 0.01)

In addition, as expected, the transcript levels of the ethylene biosynthesis genes *(DcACO1* and *DcACS1)* were also significantly higher in controls than in SNP treatments for all the cultivars (Fig. 8). Furthermore, the transcript levels of the senescence-related gene (*DcCPI*) were also higher in the SNP treatments than in the controls (Fig. 9).

**Fig. 10:**
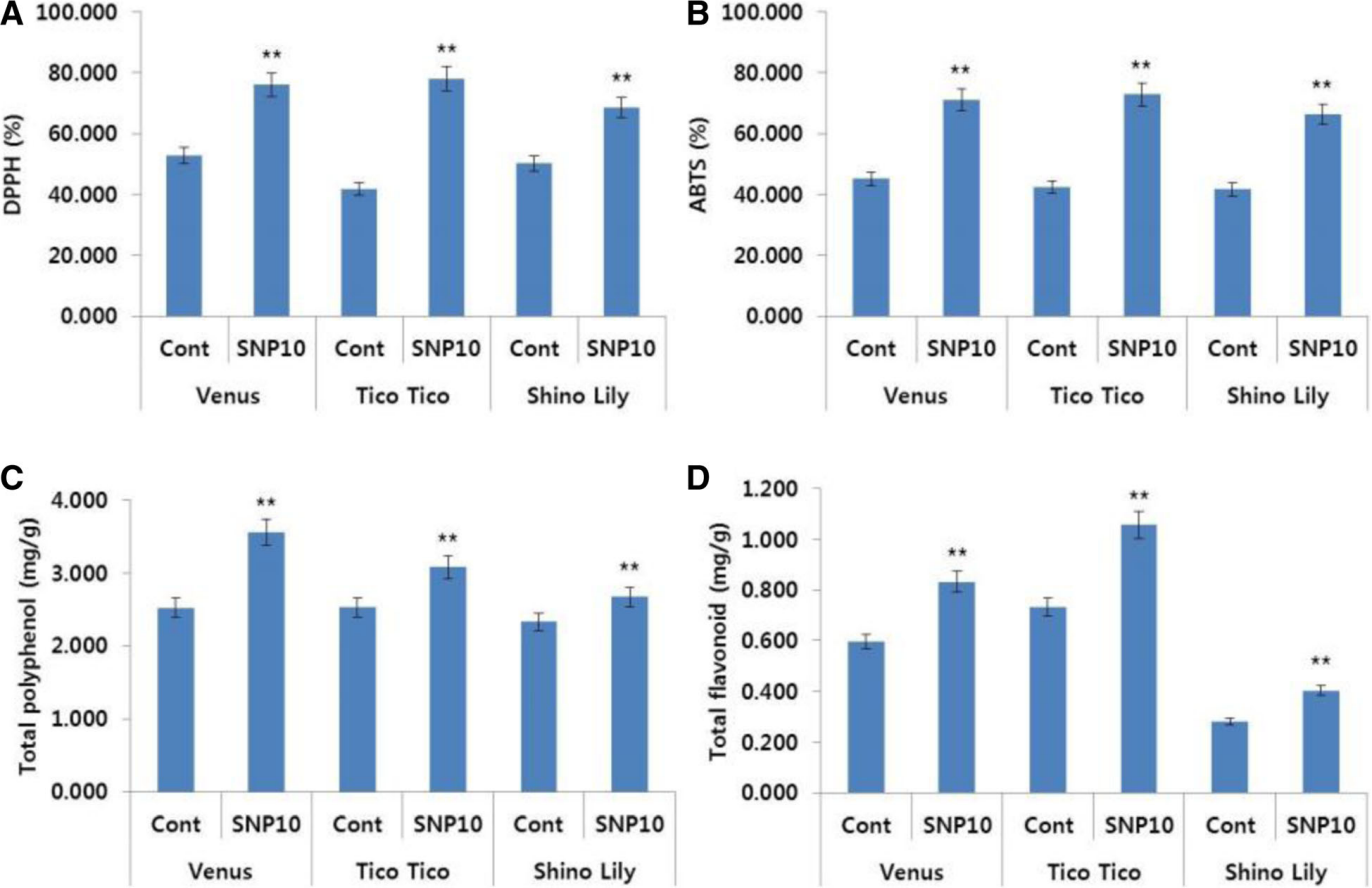
Effects of sodium nitroprusside (SNP; 10 mg L^−1^) on the DPPH activity (**a**), ABTS activity (**b**), total polyphenol content (**c**), and total flavonoid content (**d**) in the petals of different carnation ‘Venus’, ‘Tico Tico’, and ‘Shino Lily’, which were collected on day 9 after the treatment. Data represent mean of three replicates, while bar indicates standard deviation. Means with two asterisks (**) are highly significant (T-test, p < 0.01)

Significantly higher ROS-scavenging activity and antioxidant activity were also detected in the SNP treatments than in the controls, for all cultivars (Fig. 10). Therefore, the findings of the genotype experiment also lent strong support the conclusion that SNP extended the vase life of cut carnation flowers by improving all the parameters that are associated with vase life of the flowers.

## Discussion

SNP, an NO donor, has garnered considerable attention amongst researchers experimenting on the vase life of cut flowers, due to the evidence for its role in extending the vase life of various cut flowers such as rose, gladiolus, and carnations [13–15]. In these experiments, SNP could markedly improve the vase life of the cut flowers by inhibiting ethylene production [13] and promoting antioxidant activity [14, 15]. However, whether SNP participates in the physical, biochemical, and genetic mechanisms underlying the processes central to the senescence of cut flowers, especially carnations, was not examined to date. Therefore, in this study, we tried to understand the role of SNP in the mechanisms underlying the determination of vase life of cut carnations, and addressed the missing links to be appropriately incorporated for creating a representative model of the cut flower senescence.

Here, we found that SNP concentrations markedly affected the vase life of cut carnation flowers; SNP at 1 mg L^−1^ concentration could enhance the vase life compared with control, but it is likely insufficient for a substantial effect, because the concentrations higher than 1 mg L^−1^ (5 and 10 mg L^−1^) significantly promoted the vase life further. However, concentrations higher than 10 mg L^−1^ appeared to reduce the vase life, resulting in a negative effect on vase life length at 20 mg L^−1^ SNP. Dwivedi et al. [14] and Zeng et al. [15] also reported similar findings in gladiolus and carnation, and claimed that low concentrations of SNP were less effective in enhancing vase life, whereas high concentrations reduced vase life. In addition, Sankhla et al. [21] and Liao et al. [13] also reported that high concentrations of SNP could reduce the vase life of phlox and rose cut flowers below the level of controls.

Previous studies have reported that the onset of petal senescence (in-rolling) coincided with the peak of ethylene production in carnations [22, 23]. Accordingly, we noticed that the long vase life of the 10 mg L^−1^ SNP treatment was associated with low ethylene production, because the level detected for SNP 10 mg L^−1^ was the lowest; such suppression of ethylene production by SNP was also observed in previous studies [3, 13, 24]. High ethylene production rates in control and 20 mg L^−1^ SNP treatment could be due to the absence of SNP that could suppress ethylene production (in the case of controls) or the presence of high SNP concentration that could be toxic to the flowers (in the 20 mg L^−1^ SNP treatment), resulting in the shortest vase life among the treatments. Moreover, it was found that a long vase life was associated with RFW as well. Although the maximum RFW was seen at day 3 for all treatments including the control, the reduction of RFW was achieved only in 10 mg L^−1^ SNP treatment on days 6 and 9, compared with the other treatments. The positive effect of SNP on RFW has been shown in roses and carnations [12, 15]. Seyf et al. [12] revealed that the positive effect was due to the role of SNP in stomata closure, reduction of transpiration, and water loss. Similar to our experiment, Seyf et al. [12] also reported a negative effect of SNP at higher concentrations on RFW, which could be due to injury to membranes and nucleic acids [25].

Many studies have reported that NO could suppress ethylene production during the postharvest life of various cut flowers by inhibiting ethylene biosynthesis components such as ACC and ACO activity [13, 24, 26]. However, to date, the association of NO to such ethylene biosynthesis processes has not been well examined in carnations. In this study, we observed that SNP could inhibit the transcript levels of *DcACS1* and *DcACO1*, and the expression patterns of the ethylene biosynthesis genes *DcACS1* and *DcACO1* mirrored the ethylene production rate, because the transcript levels of the detected genes were observed to be the lowest in 10 mg L^−1^ SNP treatment, followed by other treatments (in the order of: 5, 1, and 15 mg L^−1^), whereas those in control and 20 mg L^−1^ SNP treatment were the highest. Satoh and Waki [27] claimed that *DcACS1* were highly expressed in carnation flowers with a normal vase life. Tanase et al. [16] also claimed that the transcript levels of *DcACS1* and *DcACO1* in carnation flowers were high on days 5 and 6 after treatment, with an increase in the ethylene production. In addition, they further claimed that the differences in long vase life among different carnation cultivars were also associated with the transcript levels of the ethylene biosynthesis genes. Recently, Ichimura and Niki [5] also reported that high transcript levels of the ethylene biosynthesis genes correlated with high ethylene production and petal senescence.

Cysteine proteinase inhibitor gene (*DcCPI*) plays an important role in the regulation of carnation petal senescence by inhibiting the cysteine proteinase gene (*DcCPase*), which leads to the decomposition of cell components and cell death during petal senescence [28–30]. In this study, the expression of *DcCPI* responded differently to the treatments; its transcript level was found to be the highest in 10 mg L^−1^ SNP treatment, followed by the other treatments (5 > 15 > 1 mg L^−1^), whereas the lowest transcript levels were noted in the control and 20 mg L^−1^ SNP. These results mirrored the vase life of the cut flower. In addition, this study also supported the finding by Tanase et al. [30], because they recently claimed that the *DcCPI* acts as a suppressor of petal senescence in two carnation cultivars, the long-life cultivar MR and the ultra-long-life cultivar 532–6.

Reduction in antioxidants was positively associated with petal senescence in chrysanthemum [31, 32], rose [33], carnation [34], and gladiolus [35, 36]. The results of our study are consistent with those of previous studies because petal senescence was associated with changing patterns of antioxidant activity. In this study, the highest antioxidant activity could be detected in the 10 mg L^−1^ SNP treatment, which significantly delayed petal senescence; thus, we assumed that SNP delayed petal senescence by maintaining the antioxidant activity, which are responsible for scavenging the reactive oxygen species that damage the cell membrane. This supports the findings of Zeng et al. [15] and Dwivedi et al. [14], because they claimed that SNP could significantly extend the vase life of carnation and gladiolus cut flowers by maintaining antioxidant activity. Comparatively, the antioxidant activity was relatively lower in the control and 20 mg L^−1^ SNP treatment on day 9. Reduction in the antioxidant activity does not seem to be induced in carnations over the critical ageing period.

The addition of ethylene precursor (ACC) has been shown to shorten the vase life of flowers and to interrupt the positive effect of the SNP, which could be explained by the higher expression level of the ethylene biosynthesis genes, and senescence-related gene transcript levels. Moreover, the antioxidant activity detected in the ACC treatment distinctly decreased compared with the control, the activity of which was lower than that of the ACC + SNP treatment. Taken together, the data presented here provide experimental evidence of the close association between ACC and petal senescence in cut flowers; in addition, the results indicated a positive role of SNP against the ethylene precursor (ACC) in extending the vase life of cut carnation flowers.

Confirmation of the effects of SNP treatment (10 mg L^−1^) on the vase life of different cultivars was also performed, using the same physiological parameters and molecular approaches, as used for Tico Viola. Here, we found that the application of SNP could increase the vase life of all the different cultivars tested via suppression of ethylene production and its related genes as well as the petal-senescence gene, and by maintaining antioxidant activity during flower development. The differences of results in response to SNP would be genotype-dependent. Therefore, the substantial confirmation of the roles of SNP presented here strongly suggests that SNP can be exploited as a novel agent for the improvement of vase life of different cut flowers.

## Conclusion

We have demonstrated that sodium nitroprusside (SNP), an NO donor, could extend the vase life of the carnation Tico Viola by significantly suppressing ethylene production and maintaining antioxidant activity, compared with the control. Moreover, it could downregulate the expression of ethylene production-related genes and petal-senescence gene. SNP at 10 mg L^−1^ concentration gave the best results among the treatments, and was still effective when it was combined with ACC. Further confirmation of the roles of SNP treatment in the vase life of different cultivars supports the results observed in Tico Viola. Taken together, it is clear that a long vase life is associated with ethylene production, antioxidant activity, ethylene production-related genes, and petal-senescence genes, and it seems that SNP is involved in multiple modes of action apart from the ethylene response during the senescence of cut carnation flowers.

## Additional file

Additional file 1: Table S1. Primer sequences used for detection of genes related to ethylene production and petal senescence by qRT-PCR. **Figure S1.** Effects of different concentrations of SNP (mg L^−1^) on the petal senescence of ‘Tico Viola’. The photo was taken on day 9 after the treatment. **Figure S2.** Effects of ACC (1 mg L^−1^) and ACC (1 mg L^−1^) + SNP10 (10 mg L^−1^) on the petal senescence in ‘Tico Viola’. The photo was taken on day 9 after the treatment. **Figure S3.** Effects of SNP (10 mg L-1) on the petal senescence of different carnation ‘Venus’, ‘Tico Tico’, and ‘Shino Lily’. Photo was taken on day 9 after the treatment. (DOCX 262 kb)

## Abbreviations

1-MCP: 1-methylcyclopropene
ABTS: 2, 2′-azino-bis-3-ethylbenzthiazoline-6-sulphonic acid
ACC: 1-aminocyclopropane-1-arboxylic acid
ACO: 1-aminocyclopropane-1-carboxylate oxidase
ACS: 1-aminocyclopropane-1-carboxylate synthase
DPPH: 1, 1-diphenyl-2-picrylhydrazyl radical
NO: Nitric Oxide
qRT-PCR: quantitative real-time polymerase chain reaction
RFW: Relative fresh weight
ROS: Reactive oxygen species
SNP: Sodium nitroprusside
STS: Silver thiosulfate
