# Supplementary material for: Characterization of the role of sodium nitroprusside (SNP) involved in long vase life of different carnation cultivars
Source: BMC Plant Biol. 2017 Sep 6;17:149. doi: 10.1186/s12870-017-1097-0 (PMC5586022; doi:10.1186/s12870-017-1097-0)
Supplement: Additional file 1: Table S1. — Primer sequences used for detection of genes related to ethylene production and petal senescence by qRT-PCR. Figure S1. Effects of different concentrations of SNP (mg L−1) on the petal senescence of ‘Tico Viola’. The photo was taken on day 9 after the treatment. Figure S2. Effects of ACC (1 mg L−1) and ACC (1 mg L−1) + SNP10 (10 mg L−1) on the petal senescence in ‘Tico Viola’. The photo was taken on day 9 after the treatment. Figure S3. Effects of SNP (10 mg L-1) on the petal senescence of different carnation ‘Venus’, ‘Tico Tico’, and ‘Shino Lily’. Photo was taken on day 9 after the treatment. (DOCX 262 kb) [file 12870_2017_1097_MOESM1_ESM.docx]

Table 1 Primer sequences used for detection of genes related to ethylene production and petal senescence by qRT-PCR

| Gene | Primer sequence (5'-3') | PCR condition |
| --- | --- | --- |
| *ACO1* | F- CCG AGC AAC TGT TGG ACT TG  R- AGA GAA TGA TGC CAC CAG CG | 95°C (10 min) → [95°C (15 s) → 57°C (1 min) →72°C (35 s)] × 40 cycles → 95°C (15 s) → 59.3°C (1 min) → 95°C (15 s) |
| *ACS1* | F- TCC AGG GTT TAG GGT TGG GA  R- CCT TCC TAC AAA CGC CTC GT |  |
| *CPI* | F- GGT GAA ACC GTG GGT GAA CT  R- CCT TCC AGA AAC ATG CTC CG |  |
| *Actin* | F- GCA CGG TAT CGT CAC CAA CT  R- AGC CTT TGG GTT AAG AGG CG |  |


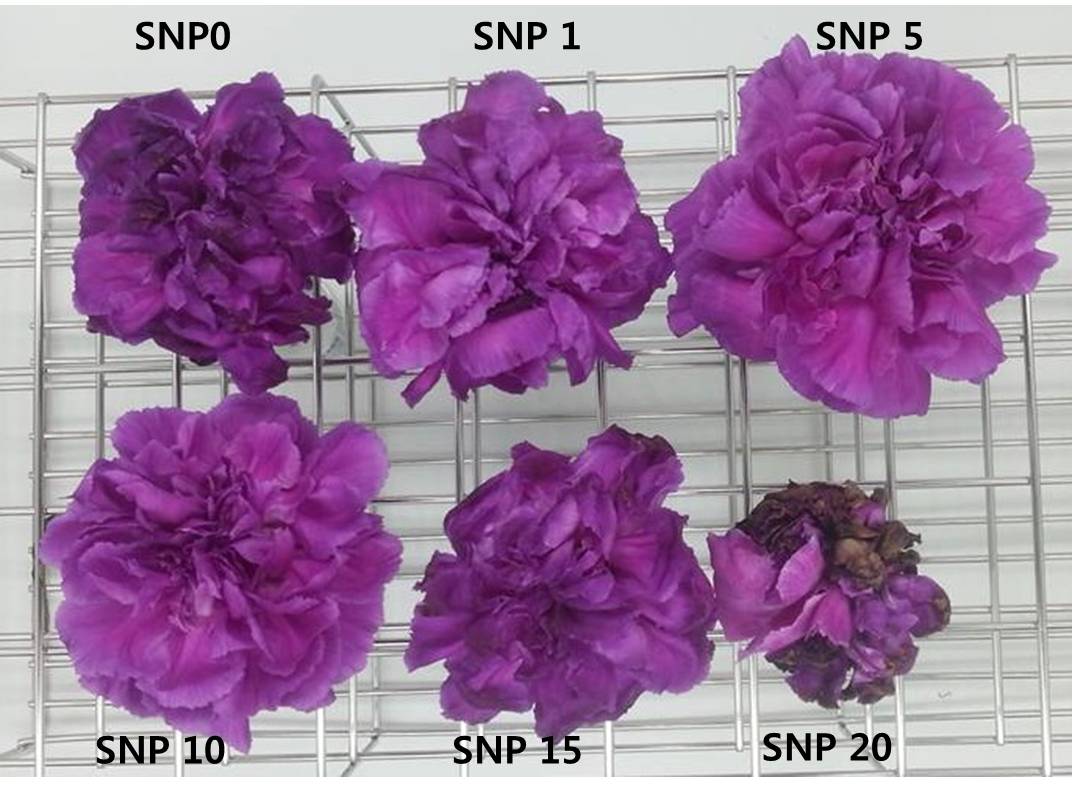


Fig. 1 Effects of different concentrations of SNP (mg L^-1^) on the petal senescence of ‘Tico Viola’. The photo was taken on day 9 after the treatment.


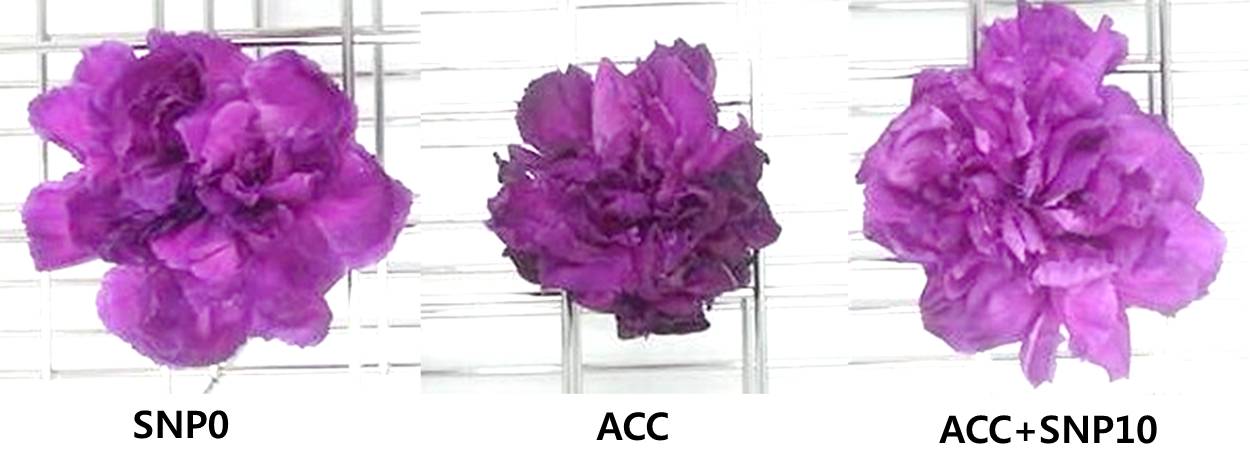


Fig. 2 Effects of ACC (1 mg L^-1^) and ACC (1 mg L^-1^) +SNP10 (10 mg L^-1^) on the petal senescence in ‘Tico Viola’. The photo was taken on day 9 after the treatment.


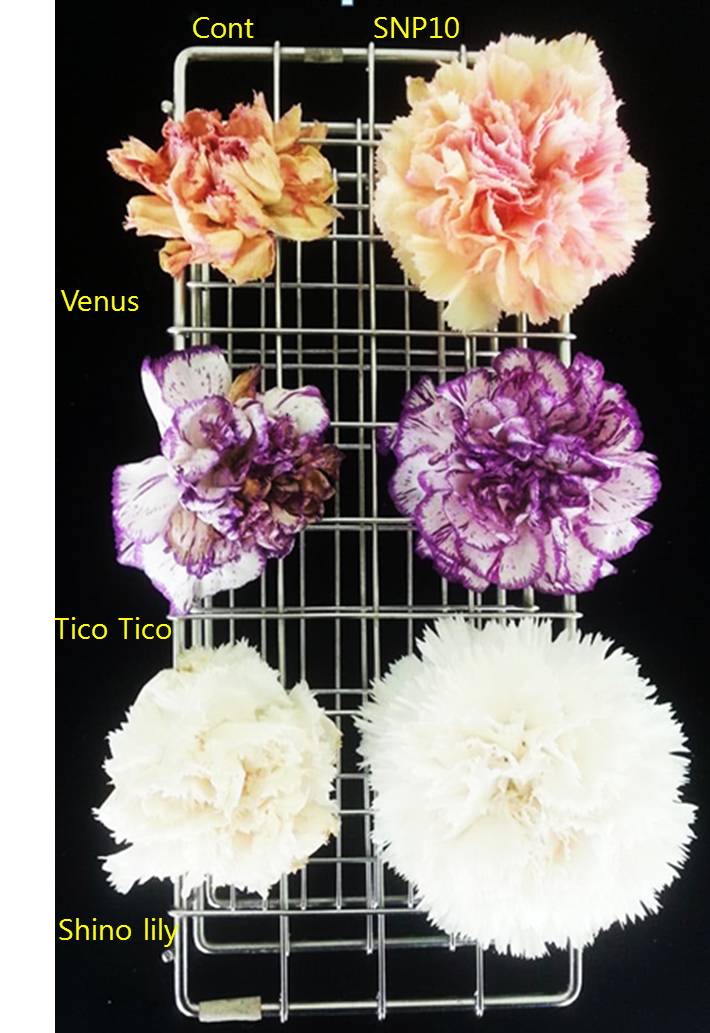


Fig. 3 Effects of SNP (10 mg L^-1^) on the petal senescence of different carnation ‘Venus’, ‘Tico Tico’, and ‘Shino Lily’. Photo was taken on day 9 after the treatment.
